# Artificial intelligence-assisted rapid on-site evaluation in liver biopsy: a diagnostic accuracy study

**DOI:** 10.3389/fonc.2026.1740247

**Published:** 2026-04-22

**Authors:** Cheng-Cheng Du, Yu-Xian Chen, Chun-Hai Li, Hong- Meng, Fan-Lei Kong

**Affiliations:** Department of Radiology, Qilu Hospital of Shandong University, Jinan, China

**Keywords:** artificial intelligence, biopsy, liver lesions, rapid on-site evaluation (ROSE), tumor

## Abstract

**Background:**

Liver biopsy is the gold standard for diagnosing liver lesions but is hampered by the time-consuming nature of conventional pathology. Artificial Intelligence-assisted Rapid On-Site Evaluation (AI-ROSE) offers a promising solution for real-time assessment of biopsy samples. This study aims to evaluate the diagnostic performance of AI-ROSE in liver biopsies using histopathology as the reference standard.

**Methods:**

Fifty-eight patients with liver lesions undergoing CT-guided percutaneous biopsy were prospectively enrolled. Each biopsy sample underwent triple assessment: AI-ROSE analysis, exfoliative cytology, and histopathological examination. Diagnostic sensitivity, specificity, accuracy, and predictive values were calculated. Serial (both tests positive) and parallel (either test positive) combinations of AI-ROSE and cytology were also analyzed. The agreement between methods was assessed using the Kappa statistic.

**Results:**

AI-ROSE showed a sensitivity of 92.31% (95% CI: 81.8% - 97.1%) and an accuracy of 87.93% (95% CI: 77.2% - 94.2%), outperforming exfoliative cytology (sensitivity: 86.54%; accuracy: 84.48%). The parallel application of AI-ROSE and cytology achieved the highest sensitivity of 96.15% (95% CI: 87.02-100.00) and significantly improved agreement with the gold standard (Kappa = 0.498, p = 0.0015).

**Conclusion:**

This study shows AI-ROSE not only has better sensitivity and accuracy in single-item diagnosis than exfoliative cytology in liver biopsy, but demonstrates excellent synergistic value in parallel application, which can greatly improve the reliability of intraoperative diagnosis. This indicates AI-ROSE has important application prospects in optimizing the clinical decision-making process and reducing the risk of intraoperative missed diagnosis.

## 1 Introduction

Liver biopsy is the gold standard for the diagnosis of liver lesions and an important tool for evaluating liver diseases (1). In the diagnostic process of liver diseases, although imaging examinations and clinical tests play indispensable roles, they also have certain limitations; therefore, liver biopsy remains an important method for diagnosing liver diseases (2). In conventional pathological examinations, it usually takes 3 to 4 days from sample collection to result reporting, and cytological tests also require at least 2 to 3 days. This prolongs hospital stays, increases medical costs, and challenges the timeliness of clinical decision-making.

In interventional examinations, the timeliness of diagnostic results is crucial for subsequent treatment. To address this issue, rapid on-site evaluation (ROSE), as a “real-time accompanying technology”, plays an important role in the field of interventional medicine. In recent years, with the wide application of artificial intelligence (AI) technology in various clinical fields, especially in the promotion of pathological diagnosis, the combination of AI and ROSE technology and its application in interventional examinations and cancer diagnosis have shown great development potential and application prospects (3, 4).

AI-ROSE technology provides a method to quickly obtain the histocytomorphological characteristics of biopsy samples to assess the degree of benignity or malignancy of the samples (5), which is of great guiding significance for subsequent treatment decisions, especially interventional treatment. Pathologists and cytologists can conduct professional evaluation and interpretation of samples, but due to resource limitations, they cannot be stationed in the operating room for a long time. Therefore, the use of AI-ROSE to evaluate the benignity or malignancy of biopsy samples can initially interpret the samples and assess their quality. Through this rapid on-site evaluation of biopsy samples, interventional specialists can quickly develop the next treatment plan after the sample is obtained, significantly shortening the waiting time for patients and providing an effective means for achieving timely and accurate treatment. This method is expected to improve the efficiency of diagnosis and treatment and shorten the patient waiting process. Therefore, this diagnostic accuracy study was conducted to rigorously evaluate the performance of a novel AI-ROSE system against the histopathological gold standard in patients undergoing liver biopsy. A secondary aim was to explore its synergistic value when combined with conventional exfoliative cytology.

## 2 Methods

### 2.1 Research subjects

**Figure 1:**
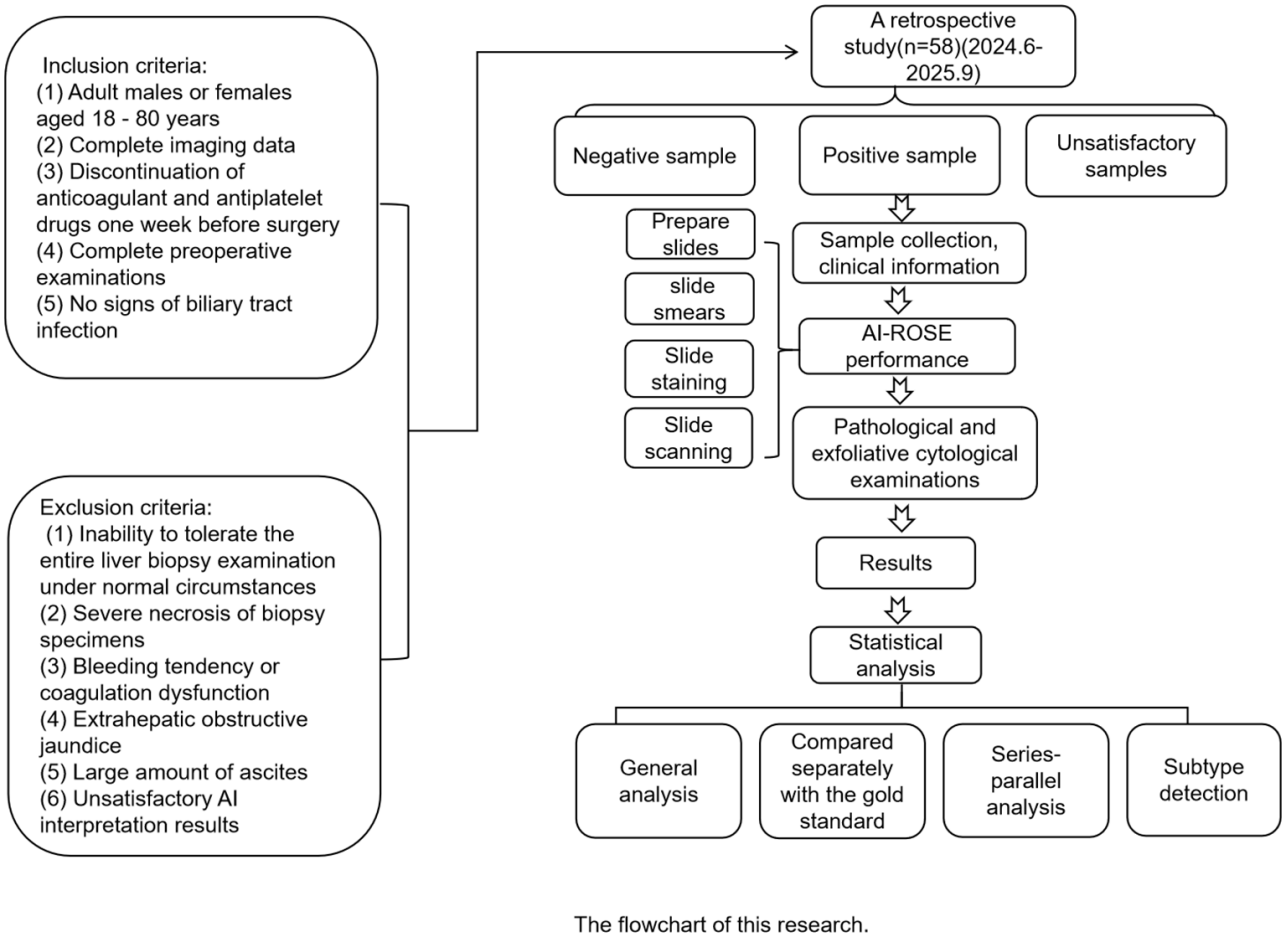
The flowchart of this research.

A total of 58 patients with liver lesions who underwent liver biopsy or combined liver biopsy and liver ablation at Qilu Hospital of Shandong University between June 2024 and September 2025 were included in this study (as shown in **Figure 1**). All enrolled patients underwent both exfoliative cytology and histopathological examination, with the histopathological results serving as the gold standard. All cases had clear pathological diagnoses and molecular cytological diagnoses. All patients signed informed consent forms. This study was approved by the Medical Ethics Review Committee of Qilu Hospital of Shandong University (No.:KYLL-202408-026). Inclusion criteria include: (1) Adult males or females aged 18–80 years; (2) Complete imaging data, with abdominal CT or PET-CT and other imaging examinations indicating liver space-occupying lesions, or clinically highly suspected liver neoplastic lesions; (3) Discontinuation of anticoagulant and antiplatelet drugs one week before surgery; (4) Complete preoperative examinations such as electrocardiogram, blood routine, coagulation function, HIV, and hepatitis markers before surgery; (5) No signs of biliary tract infection. Exclusion criteria include: (1) Inability to tolerate the entire liver biopsy examination under normal circumstances; (2) Severe necrosis of biopsy specimens; (3) Bleeding tendency or coagulation dysfunction; (4) Extrahepatic obstructive jaundice; (5) Large amount of ascites; (6) Unsatisfactory AI interpretation results.

### 2.2 Study methods and sample

Percutaneous liver biopsies were performed under CT guidance using a standard clinical protocol. Following local anesthesia, one to two core tissue samples were obtained with an automated biopsy needle. Immediately after retrieval, the fresh biopsy core was gently and meticulously rolled onto a sterile glass slide to create a touch imprint smear for AI-ROSE analysis. The residual tissue was fixed in formalin for routine histopathological processing, which served as the reference standard. All the biopsy procedures and slide preparations were carried out by interventional physicians with more than 10 years of experience.

The air-dried smear was rapidly stained using the Diff-Quik kit, following the manufacturer’s protocol. Briefly, the slide was sequentially immersed in Solution A and Solution B, with intermediate PBS rinses to ensure clarity. After drying, the slide was digitized using a dedicated microscopic image analysis system (Shanghai Xingmai Information Technology Co., Ltd.) for automated AI interpretation. Following the AI-ROSE assessment, the same slide was submitted to the cytology department for independent evaluation by a cytopathologist, who was blinded to the AI result. The experimental materials, equipment, and reagents are detailed in **Table 1**.

**Table 1:** Experimental materials, equipment, and reagents.

Experimental materials and equipment	Model (Specification)	Manufacturer
Digital Microscopy Image Analysis System	AT1	Fosun Xingmai(China)
Diff-Quik Staining Solution A	BA-4100	BASO(China)
Diff-Quik Staining Solution B	BA-4100	BASO(China)
Phosphate buffered solution	500ml	BASO(China)
ROSE Customized Microscope Slides	26mm*76mm	Fosun Xingmai(China)

### 2.3 Principle of AI-ROSE

**Figure 2:**
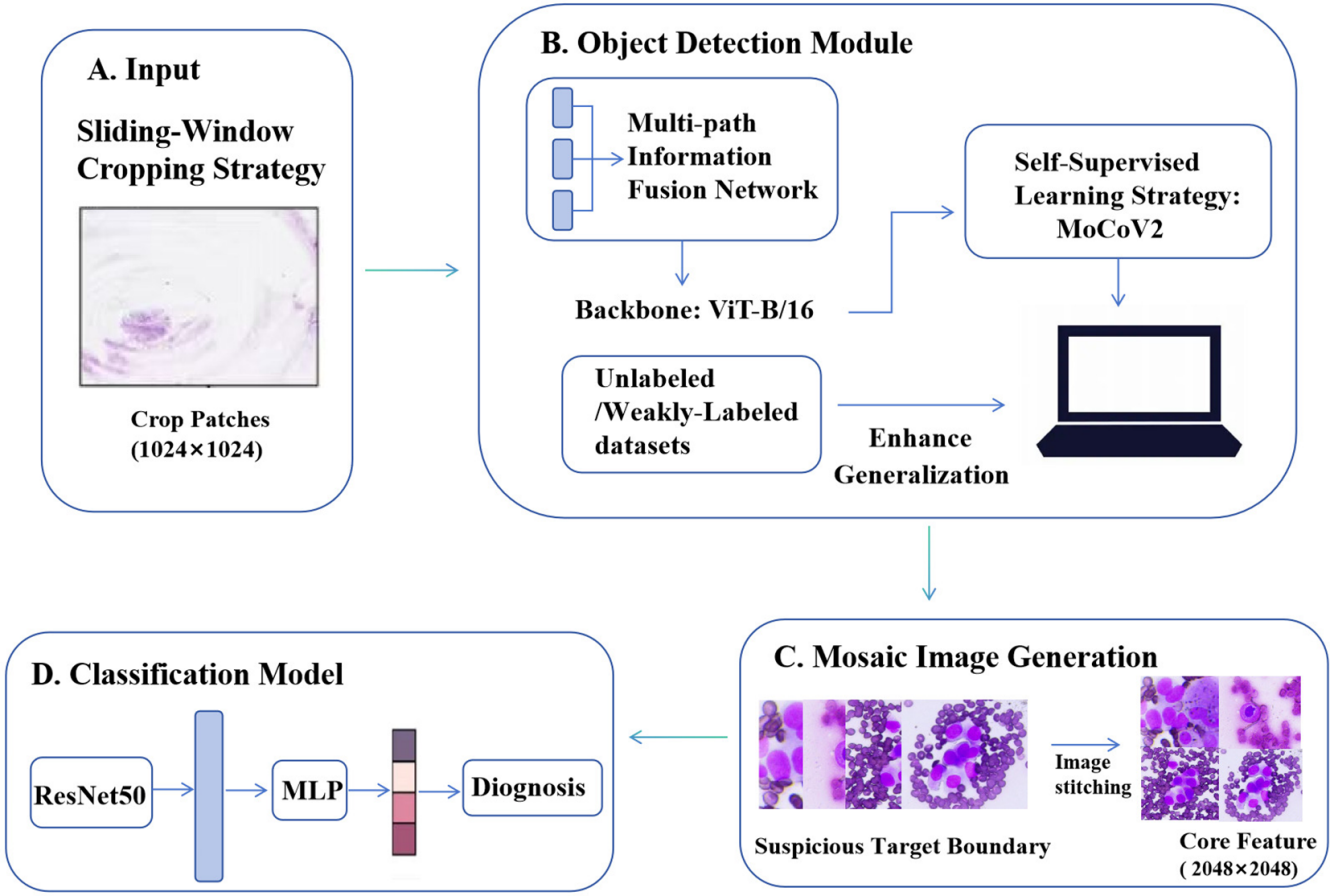
The Principle of AI-ROSE: A two-stage progressive architecture with an embedded multi-step attention enhancement mechanism. **(A–C)**: front-end object detection module; **(D)**: back-end classification module.

The AI ROSE intelligent analysis model proposed in this study adopts a two-stage progressive architecture with an embedded multi-step attention enhancement mechanism (overall structure shown in **Figure 2**). The model consists of two core components: a front-end object detection module (workflow illustrated in **Figures 2A–C**) and a back-end classification module (workflow illustrated in **Figure 2D**), which together perform intelligent analysis of whole-slide cytological images.

For large-sized full-section cytological images, a sliding window cropping strategy is employed to divide the original slices into standard image blocks with a resolution of 1024×1024, and these blocks are input into the detection model in batches (as shown in **Figure 2A**). The object detection module employs a multi-path feature fusion network as its core architecture, with ViT-B/16 selected as the backbone for feature extraction. Its main function is to accurately distinguish normal tissue from diseased tissue, enabling the localization and identification of abnormal cells (as shown in **Figure 2B**). To enhance the feature generalization capability of the core network, this module innovatively introduces the MoCoV2 self-supervised learning strategy, pre-training on unlabeled or weakly labeled datasets, thereby effectively alleviating the scarcity of annotated cytological data (**Figure 2B**). Following the above processing, the module outputs bounding box coordinates corresponding to the abnormal cells.

Based on the detected bounding boxes, the model automatically extracts highly suspicious lesion regions, and multiple independent suspected abnormal regions are integrated into a single mosaic image of 2048×2048 resolution using an image stitching algorithm, achieving a concentrated and compact representation of suspicious lesion features (workflow illustrated in **Figure 2C**). This mosaic image serves as the core feature input to the subsequent classification module for final diagnosis.

The classification module uses a lightweight ResNet50 network as the backbone feature extractor, with its lightweight design balancing inference efficiency and feature extraction accuracy. This is followed by a multi-layer perceptron (MLP) to perform binary classification (benign vs. malignant) of the pathology slides (as shown in **Figure 2D**).

### 2.4 Quality control

Smear preparation: During the procedure, repeated smearing over the same area was avoided to prevent excessive cell stacking, which could interfere with the clear visualization of single-cell morphology. Each specimen was prepared using a gentle, unidirectional, and uniform smearing technique to ensure even cell distribution and a monolayer flat spread.

Staining time control: The staining time of the Diff-Quik protocol was flexibly adjusted according to the cell density on the smear. For cell-dense smears, the staining time was appropriately shortened; for cell-sparse smears, it was prolonged accordingly. This approach maintained optimal nuclear-cytoplasmic contrast and avoided interpretation errors caused by over-staining or under-staining.

Region selection for AI-ROSE reading: Prior to automatic AI-ROSE interpretation, the operator manually identified regions with the highest cellular quality under a microscope (e.g., areas with intact cell morphology, clear staining, and no overlapping or debris). Within these regions, the number of AI reading points was increased to enhance the representativeness and accuracy of the interpretation.

Multi-point simultaneous sampling: For each patient, at least two cytology smears were prepared simultaneously during the same puncture procedure. If one smear exhibited insufficient or poor-quality cellular material, the remaining smears could be used for interpretation. Furthermore, consistent results across multiple smears further reduced the risk of false negatives. This strategy effectively minimized the impact of sampling error from a single smear on the diagnostic outcome.

### 2.5 Sample interpretation

**Figure 3:**
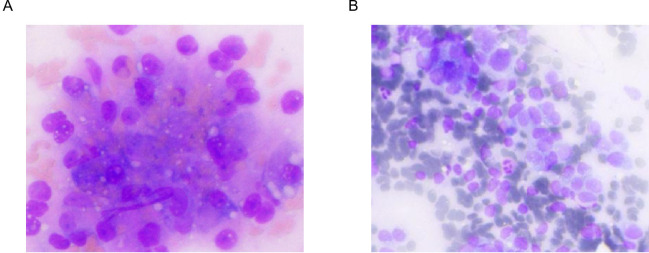
**(A)** Hepatocellular carcinoma: The cells have a distinct pleomorphism, which can be polygonal, similar to normal liver cells, or may present as spindle-shaped, giant cells, etc. The cell volume is usually large, with abundant cytoplasm and vacuoles. The nucleus is large, deeply stained, and has obvious atypia. Pathological nuclear division figures are common. **(B)** Liver Undifferentiated Carcinoma: Cells lose their classic structural arrangement. Cell size and shape vary greatly, appearing elongated and resembling fibroblasts. While some cells retain distinct nucleoli, these may be indistinct in undifferentiated cells.

The AI-ROSE analysis was performed using an artificial intelligence cytopathological diagnosis system (Shanghai Xingmai Information Technology Co., Ltd.). Digitized whole-slide images of the ROSE smears were automatically analyzed by the system’s proprietary algorithm, which integrates deep learning architectures including convolutional neural networks. The model was designed to identify malignant cells based on predefined quantitative morphometric parameters. The primary diagnostic criteria included: (1) nuclear/cytoplasmic ratio (N/C) > 0.5; (2) nucleolar to nuclear diameter ratio (n/N) > 0.25; and (3) the presence of three or more nucleoli. A case was adjudicated as AI-ROSE positive if the system identified cells meeting these criteria, indicating the presence of malignant or suspicious malignant cells. Otherwise, it was classified as AI-ROSE negative. All AI-ROSE results were recorded alongside relevant clinical data (as shown in **Figure 3**).

### 2.6 Statistical methods

The statistical analysis of this study mainly included four parts: analysis of general patient information; comparative analysis of AI-ROSE and exfoliative cytology with histopathology (gold standard) respectively; analysis of combined tests (serial test: positive only when both methods are positive; parallel test: positive when either method is positive); and subgroup analysis of patients with different subtypes of liver cancer. SPSS 25.0 software was used for statistical analysis. Sensitivity, specificity, positive predictive value (PPV), and negative predictive value (NPV) were calculated to evaluate the diagnostic accuracy of AI-ROSE and conventional pathological biopsy. Kappa test was used for consistency analysis: Kappa > 0.8 indicates excellent consistency, 0.6 - 0.8 indicates high consistency, 0.4 - 0.6 indicates moderate consistency, and Kappa < 0.4 indicates weak consistency. McNemar test was used to analyze the distribution trend of inconsistencies. P-value < 0.05 was considered statistically significant.

## 3 Study results

### 3.1 General information

A total of 58 liver tissue samples were collected in this study, including 37 males and 21 females, with an average age of 59 years. The demographic characteristics are shown in **Table 2**. AI-ROSE indicated 9 cases of benign lesions and 49 cases of malignant lesions; cytology showed 11 negative cases and 47 positive cases; pathological results indicated 6 benign cases and 52 malignant cases. Among the malignant lesions, there were 21 cases of hepatocellular carcinoma, 11 cases of cholangiocellular carcinoma, 1 case of mixed hepatocellular-cholangiocellular carcinoma, and 19 cases of metastatic liver cancer. The characteristics of patients’ liver lesions and biopsy results are shown in **Table 2**.

**Table 2:** Demographic characteristics of 58 patients.

Variable	No. of patients (%)
Age (years).mean ± SD	59.34 ± 13.76
Size of lesions(cm).mean ± SD	6.37 ± 4.43
Sex	
Male	37(63.79%)
Female	21(36.21%)
Location	
Left lobe of liver	11(18.97%)
Right lobe of liver	47(81.03%)
The location of the primary tumor lesion	
No tumor lesion	6(10.34%)
Tumor of liver	33(56.90%)
Tumors in other parts	19(32.76%)
Abnormal liver function	46(79.31%)
History of hepatitis	33(56.90%)
History of liver cirrhosis	16(27.59%)
Abnormal AFP value	23(39.66%)
Abnormal CEA value	17(29.31%)
Abnormalities in other tumor markers	33(56.90%)
History of smoking	23(39.66%)
Drinking history	24(41.38%)

### 3.2 AI-ROSE interpretation results and consistency with pathological biopsy

Among the 58 patients, 49 were positive and 9 were negative by AI-ROSE diagnosis. Pathological results indicated 6 benign cases and 52 malignant cases. Compared with the pathological gold standard, the accuracy of AI-ROSE in diagnosing liver lesions was 87.93% (95% CI: 77.2% - 94.2%), sensitivity was 92.31% (95% CI: 81.8% - 97.1%), specificity was 50.00% (95% CI: 18.8% - 81.2%), positive predictive value was 94.12% (95% CI: 83.8% - 98.1%), negative predictive value was 42.86% (95% CI: 16.8% - 73.6%), and Youden index was 42.31% (see **Table 3**). Compared with conventional pathological biopsy, AI cytopathological diagnosis had a good correlation with histopathological diagnosis results, which was statistically significant, and there was no significant tendency in the distribution of inconsistent parts (McNemar test: χ² = 0, p > 0.99). In addition, the consistency between AI cytopathological diagnosis and histopathological diagnosis results was low (Kappa = 0.394, p = 0.067) (see **Table 3** for details).

**Table 3:** AI-ROSE diagnosis vs. pathological gold standard.

Diagnosis	Pathology: Positive (1)	Pathology: Negative (0)	Total
AI: Positive (1)	TP=48	FP=3	51
AI: Negative (0)	FN=4	TN=3	7
Total	52	6	N=58

TP, True Positive; FP, False Positive; FN, False Negative; TN, True Negative.

### 3.3 Consistency between exfoliative cytology and pathological biopsy

Exfoliative cytology showed 11 negative cases and 47 positive cases; pathological results indicated 6 benign cases and 52 malignant cases. Compared with the pathological gold standard, the accuracy of exfoliative cytology in diagnosing liver lesions was 84.48% (95% CI: 73.5% - 91.6%), sensitivity was 86.54% (95% CI: 76.2% - 92.8%), specificity was 66.67% (95% CI: 35.0% - 88.5%), positive predictive value was 95.74% (95% CI: 85.9% - 98.8%), negative predictive value was 36.36% (95% CI: 18.5% - 59.1%), and Youden index was 53.21% (see **Table 4**). Compared with conventional pathological biopsy, AI cytopathological diagnosis had a significant correlation with histopathological diagnosis results, and the difference between them was not statistically significant (McNemar test: χ² = 1.778, p = 0.182). In addition, the consistency between exfoliative cytology diagnosis and histopathological diagnosis results was significantly higher than that by chance (Kappa = 0.397, p < 0.001).

**Table 4:** Exfoliative cytology vs. pathological gold standard.

Diagnosis	Pathology: Positive (1)	Pathology: Negative (0)	Total
Cytology: Positive (1)	TP=45	FP=2	47
Cytology: Negative (0)	FN=7	TN=4	11
Total	52	6	N=58

### 3.4 AI-ROSE interpretation results and serial diagnosis with cytology vs. pathological diagnosis

Among the 58 patients, 43 were positive and 15 were negative by serial diagnosis. Compared with the pathological gold standard, the diagnostic accuracy of serial diagnosis was 77.59% (95% CI: 65.5% - 86.5%), sensitivity was 78.85% (95% CI: 66.2% - 87.8%), specificity was 66.67% (95% CI: 29.9% - 89.7%), positive predictive value was 95.35% (95% CI: 83.8% - 98.8%), negative predictive value was 26.67% (95% CI: 11.8% - 49.3%), and Youden index is shown in **Table 5**. Compared with conventional pathological biopsy, the serial diagnosis had a significant correlation with histopathological diagnosis results, and the difference between them was statistically significant (McNemar test: χ² = 4.923, p = 0.027). In addition, the consistency between exfoliative cytology diagnosis and histopathological diagnosis results was significantly higher than that by chance (Kappa = 0.274, p < 0.05).

**Table 5:** Serial diagnosis (AI-ROSE + exfoliative cytology) vs. pathological gold standard.

Diagnosis	Pathology: Positive (1)	Pathology: Negative (0)	Total
Serial diagnosis positive results (1)	41	2	43
Serial diagnosis negative results (0)	11	4	15
Total	52	6	58

### 3.5 AI-ROSE interpretation results and parallel diagnosis with cytology vs. pathological diagnosis

Among the 58 patients, 53 were positive and 5 were negative by parallel diagnosis. Compared with the pathological gold standard, the diagnostic accuracy of parallel diagnosis was 91.38% (95% CI: 81.38% - 96.26%), sensitivity was 96.15% (95% CI: 87.02% - 100.00%), specificity was 50% (95% CI: 18.76% - 81.24%), positive predictive value was 94.34% (95% CI: 84.62% - 98.05%), negative predictive value was 60% (95% CI: 23.07% – 88.24%), and Youden index was 46.15% (see **Table 6**). Compared with conventional pathological biopsy, the parallel diagnosis had a significant correlation with histopathological diagnosis results, and the difference between them was not statistically significant (McNemar test: χ² = 0, p = 1). In addition, the consistency between exfoliative cytology diagnosis and histopathological diagnosis results was significantly higher than that by chance (Kappa = 0.498, p < 0.0015).

**Table 6:** Parallel diagnosis (AI-ROSE + exfoliative cytology) vs. pathological gold standard.

Diagnosis	Pathology: Positive (1)	Pathology: Negative (0)	Total
Parallel diagnosis positive results (1)	50	3	53
Parallel diagnosis negative results (0)	2	3	5
Total	52	6	58

### 3.6 Consistency between AI-ROSE and exfoliative cytology

Among the 58 patients, 49 were positive and 9 were negative by AI-ROSE; exfoliative cytology showed 11 negative cases and 47 positive cases. A total of 48 patients had consistent diagnoses by AI-ROSE and exfoliative cytology, with an accuracy rate of 82.76% (95% CI: 70.5% - 90.8%), including 43 cases of both positive and 5 cases of both negative. In addition, 4 patients were judged negative by AI-ROSE but positive by exfoliative cytology; 6 patients were judged negative by exfoliative cytology but positive by AI-ROSE (see **Table 7**). Compared with exfoliative cytology, AI cytopathological diagnosis had a significant correlation with exfoliative cytology diagnosis results, but there was no significant difference in the inconsistent parts (McNemar test: χ² = 0.1 (after correction), p = 0.752). In addition, there was a significant correlation between AI cytopathological diagnosis and exfoliative cytology diagnosis results, but the consistency was weak (Kappa = 0.397, p = 0.022).

**Table 7:** AI-ROSE vs. exfoliative cytology.

Diagnosis	Cytology: Positive (1)	Cytology: Negative (0)	Total
AI: Positive (1)	TP=43	FP=6	49
AI: Negative (0)	FN=4	TN=5	9
Total	47	11	N=58

### 3.7 Comparison between AI-ROSE and exfoliative cytology in diagnosing primary malignant liver tumors

Among 33 patients, AI-ROSE showed 30 positive cases and 3 negative cases; exfoliative cytology showed 28 positive cases and 5 negative cases. A total of 27 patients had consistent diagnoses by AI-ROSE and exfoliative cytology, with the accuracy rate shown in **Table 8**, including 26 cases of both positive and 1 case of both negative. In addition, 2 patients were judged negative by AI-ROSE but positive by exfoliative cytology; 4 patients were judged negative by exfoliative cytology but positive by AI-ROSE (see **Table 8**). The correlation test results of the two methods showed that the correlation and consistency between them were weak (Kappa = 0.155, p > 0.05), and the difference was not statistically significant (McNemar test: χ² = 0.167, p > 0.05).

**Table 8:** AI-ROSE vs. exfoliative cytology in diagnosing primary malignant liver tumors.

Diagnosis	Cytology: Positive (1)	Cytology: Negative (0)	Total
AI: Positive(1)	TP=26	FP=4	30
AI: Negative(0)	FN=2	TN=1	3
Total	28	5	N=33

A total of 58 patients undergoing liver biopsy were included in this study. Using pathological examination as the gold standard, the diagnostic efficacy of AI-ROSE, exfoliative cytology, and their serial and parallel protocols was compared. At the same time, the time required for each method to obtain results was evaluated. The results showed that the parallel combination of AI-ROSE and exfoliative cytology had the highest sensitivity (96.15%, 95% CI: 87.02% - 100.00%) and the highest consistency with the gold standard (Kappa = 0.498, p = 0.0015). In addition, AI-ROSE was superior to exfoliative cytology in terms of sensitivity (92.31%, 95% CI: 81.8% - 97.1%) and accuracy (87.93%, 95% CI: 77.2% - 94.2%). A direct comparison of AI-ROSE and exfoliative cytology in diagnosing liver diseases showed that they had a significant correlation, but the diagnostic consistency was moderate (Kappa= 0.397), and the difference in inconsistent cases was not statistically significant (χ² = 0.1, p = 0.752). The correlation and consistency between the two methods in diagnosing primary liver cancer were both weak. The data from this study shows that the average time for AI-ROSE is 251.60 ± 13.88 seconds, while for histopathology it is 2.67 ± 1.42 days, and for cytology it is 1.38 ± 0.86 days.

## 4 Discussion

mLiver biopsy is the most specific examination for evaluating the nature and severity of different liver diseases (6), and also an important method for guiding liver ablation and liver particle implantation. With the continuous advancement of laboratory tests and imaging technology, non-invasive evaluation methods have gradually emerged, which have greatly reduced the need for liver biopsy (7). In addition, some non-invasive examination methods have also shown good results in the staging of liver diseases (8). However, despite the significant progress of non-invasive technologies, histopathological evaluation of liver diseases is still indispensable in certain specific clinical scenarios (7, 9, 10). Especially in patients with liver masses of unknown etiology identified by radiological imaging, liver biopsy is of great significance (11).

The core finding of this study is that AI-ROSE outperforms exfoliative cytology in terms of sensitivity (92.31%, 95% CI: 81.8% - 97.1%), accuracy (87.93%, 95% CI: 77.2% - 94.2%), and consistency with the pathological gold standard (Kappa = 0.394, p = 0.067). Furthermore, after parallel application of the two methods, the comprehensive sensitivity was further increased to 96.15% (95% CI: 87.02% - 100.00%), and the consistency with the gold standard was also significantly improved (Kappa = 0.498, p = 0.0015), showing a strong complementary effect. This helps to significantly reduce the risk of repeated puncture caused by insufficient sampling, indicating that AI-ROSE has good positive diagnostic efficacy. In addition, although there is a significant correlation between AI-ROSE and exfoliative cytology, the diagnostic consistency between them is only moderate (Kappa = 0.397), and the distribution of inconsistent cases has no statistical difference (χ² = 0.1 (after correction), p = 0.752), which further confirms that the two methods have different focuses in diagnostic bases and interpretation logic, thus forming an effective complement in combined application. Compared with conventional pathological biopsy, AI-ROSE has the advantages of speed and real-time performance, which can significantly shorten the waiting time for patients, and is especially suitable for rapid intraoperative judgment of lesion nature (such as benign or malignant) or monitoring of disease progression (12).

This study shows that, compared with other diagnostic methods, AI-ROSE demonstrates a significant time advantage. The fundamental reason is that it eliminates traditional procedures such as fixation, mounting, and tissue embedding, relying instead on automated analysis for interpretation. This technical feature enables a substantial reduction in diagnostic time, while the results obtained remain highly reliable and meet the demands of real-time clinical decision-making.

The purpose of this study is to evaluate the consistency between AI-ROSE and standard pathological diagnosis to verify its clinical application potential. The higher the consistency between them, the more interventional physicians can rely on the real-time feedback results of AI-ROSE during surgery. A study by Tondo P et al. showed that when AI-ROSE indicates that the sampling is qualified, physicians can confidently terminate the puncture operation, thereby effectively avoiding repeated puncture due to insufficient samples and optimizing the diagnosis and treatment process (13–15). Moreover, in the interventional diagnosis and treatment of liver cancer, the use of AI-ROSE for real-time evaluation of puncture samples helps doctors adjust subsequent treatment strategies in real time based on the results. The incidence of liver cancer is relatively high in China (16, 17), and common treatment methods include surgical resection, chemotherapy, liver ablation, hepatic artery embolization, and liver particle implantation (18), many of which rely on pathological diagnosis or even genetic testing results. The use of AI-ROSE for rapid identification of initially screened positive cases can achieve early triage of patients and treatment selection (such as whether to perform ablation immediately), which is conducive to improving the efficiency of diagnosis and treatment. However, AI-ROSE does not replace pathology. Its value lies in its timeliness rather than in the depth of molecular analysis. In clinical practice, we advocate that AI-ROSE and pathology should complement each other: the former provides reliable intraoperative assessment of benign and malignant conditions, while the latter subsequently provides comprehensive information to guide precise treatment. In conclusion, AI-ROSE can optimize the intraoperative workflow and evaluation strategy, ensuring high sensitivity while maintaining predictive accuracy. It provides an efficient and practical auxiliary tool for the interventional diagnosis and treatment of liver cancer, holding significant value for clinical promotion.

However, AI-ROSE also has certain limitations, such as high requirements for the quality of cell smears, which is easily affected by the operator’s experience, and it is difficult to provide complete information on tissue structure. And final confirmation still needs to rely on conventional pathological examination (13). However, due to resource constraints, it is difficult to ensure that pathologists are present at all times during the surgery (19, 20). It is worth noting that current studies have shown that well-trained interventional physicians can perform AI-ROSE, but this has not been widely popularized yet (21). To improve the diagnosis rate, interventional doctors can be trained to enhance their experience (20). When conducting AI-ROSE evaluation, interventional doctors should pay additional attention to the quality of cell smears. For example, poor fixation, thick smears, and slide contamination can all affect the accuracy of result interpretation (22, 23). This study also has a limitation of selection bias, namely a relatively high proportion of malignant nodules. Therefore, future prospective studies based on real-world populations are necessary to further evaluate the predictive performance of AI-ROSE in the general population. In addition, the sample size of this study is limited, which may preclude adequate subgroup analyses. To overcome this limitation, we plan to increase the sample size in subsequent multicenter studies and prospectively evaluate the diagnostic performance in various subgroups. Nevertheless, using AI-ROSE as a screening tool can still significantly save diagnosis time, which is beneficial to patients’ postoperative recovery and reduces hospitalization costs (12, 13).

AI-ROSE technology combines the advantages of cytology and pathology (24), and can real-time evaluate the adequacy and lesion nature of samples during liver biopsy, further improving the diagnostic efficiency (25). The advantages of this technology, such as speed and automation, provide important support for its promotion and application in scenarios where pathologist resources are relatively limited. Although conventional cytological examinations (such as ordinary smears) are widely used for screening, they have certain risks of false negatives or false positives due to the limited diagnostic information they provide. In recent years, with the development of liquid-based cytology, cell block, and other preparation technologies, cytological samples can provide more abundant morphological and structural information (26, 27). On this basis, AI-ROSE not only inherits the advantages of speed and minimal invasiveness of cytological examinations, but also can rely on the processed information for in-depth judgment, effectively making up for the limitation of insufficient diagnostic depth of conventional cytology. Therefore, AI-ROSE is expected to become an important auxiliary tool in liver biopsy, especially providing real-time and reliable decision-making references for clinical practice when pathologists cannot be present in real time.

Regarding the safety considerations of this technology, in our research sample, all 58 patients underwent a single biopsy procedure and no major adverse events occurred. Postoperatively, 5 patients experienced mild and tolerable abdominal pain, and 6 patients experienced mild needle tract bleeding. These situations did not affect the surgical process and in most cases, no special treatment was required. AI-ROSE itself does not increase the risk of puncture and may reduce it, owing to its ability to provide real-time diagnosis during the procedure (28). This is particularly beneficial for patients with poor physical condition or those at high risk for conventional biopsy. By rapidly interpreting intraoperative cytology results, physicians can decide to terminate the puncture procedure when appropriate, thereby avoiding invasive procedures in high-risk patients. For nodules with atypical imaging findings, repeat sampling may be necessary on traditional biopsies if the first attempt at biopsy is inadequate. AI-ROSE provides real-time feedback on sample quality, allowing the operator to collect additional samples immediately, potentially avoiding a second procedure and reducing cumulative risk. Future prospective studies are needed to systematically evaluate the benefits of AI-ROSE in high-risk populations.

As a preliminary exploratory study, this work demonstrates the feasibility and value of AI-ROSE in interventional diagnosis and treatment. Future research directions include: (1) multicenter collaboration to expand the sample size; (2) subgroup analyses by different pathological types (e.g., hepatocellular carcinoma, intrahepatic cholangiocarcinoma, and metastatic liver cancer) to evaluate the diagnostic performance of AI-ROSE across various tumor types; (3) optimization of the AI algorithm and exploration of multimodal models integrating clinical features and imaging information to further improve diagnostic performance; and (4) prospective randomized controlled trials comparing the AI-ROSE-assisted strategy with conventional strategies in terms of diagnostic accuracy, procedural safety, and health economics.

## 5 Conclusion

This study confirms that AI-ROSE technology is superior to traditional exfoliative cytology in terms of sensitivity, accuracy, and consistency with the gold standard, and is the core of constructing an efficient diagnostic protocol. The parallel strategy based on AI-ROSE can achieve extremely high diagnostic sensitivity, which has great clinical promotion value. AI-ROSE is expected to become a new standard for rapid intraoperative pathological diagnosis.

## Data Availability

The original contributions presented in the study are included in the article/**Supplementary Material**. Further inquiries can be directed to the corresponding author.
