# Supplementary material for: Artificial intelligence-assisted rapid on-site evaluation in liver biopsy: a diagnostic accuracy study
Source: Front Oncol. 2026 Apr 22;16:1740247. doi: 10.3389/fonc.2026.1740247 (PMC13143661; doi:10.3389/fonc.2026.1740247)
Supplement: Supplementary file 1 [file DataSheet1.pdf]

Dimension	AI-ROSE	Traditional histopathology
**Main objective**	Intraoperative immediate qualitative assessment (benign/malignant)	Diagnosis, Molecular Typing, Treatment Guidance
**Timeliness**	Minute-level (intraoperative real-time)	Several days (after the operation for sample collection)
**Output information**	Cell morphology assessment (benign vs. malignant)	Organizational structure, immunohistochemistry, gene mutations, protein expression, etc.
**Clinical decision-making**	Intraoperative decision-making (whether to perform ablation, whether to terminate the puncture, whether to conduct additional biopsy)	Subsequent comprehensive treatment decisions (surgical approach, selection of TACE drugs, targeted/immunotherapy regimens)
**Advantages**	Timeliness advantage, avoiding repeated punctures and reducing patients' anxiety during waiting time	Molecular information advantage, essential for precise treatment
**Complementary relationship**	Fast screening, Intraoperative navigation	Diagnosis basis, Treatment blueprint

Supplementary Table 1: AI-ROSE provides reliable intraprocedural benign/malignant assessment, while histopathology and biomarker analysis subsequently supply the complete information required for precision treatment.
