# Supplementary material for: Artificial intelligence-assisted rapid on-site evaluation in liver biopsy: a diagnostic accuracy study
Source: Front Oncol. 2026 Apr 22;16:1740247. doi: 10.3389/fonc.2026.1740247 (PMC13143661; doi:10.3389/fonc.2026.1740247)
Supplement: Supplementary file 2 [file DataSheet2.pdf]

Treatment	No. of patients (%)
Ablation	5(8.62%)
Radioactive particle implantation procedure	5(8.62%)
TACE	26(44.83%)
Chemotherapy combined with immunotherapy	3(5.17%)
Targeted combined chemotherapy and immunotherapy	5(8.62%)
Chemotherapy	10(17.24%)
Supportive treatment	4(6.90%)

Supplementary Table 2: Short-term treatment information.
